# *LMNA* Sequences of 60,706 Unrelated Individuals Reveal 132 Novel Missense Variants in A-Type Lamins and Suggest a Link between Variant p.G602S and Type 2 Diabetes

**DOI:** 10.3389/fgene.2017.00079

**Published:** 2017-06-15

**Authors:** Alyssa Florwick, Tejas Dharmaraj, Julie Jurgens, David Valle, Katherine L. Wilson

**Affiliations:** ^1^Department of Cell Biology, Johns Hopkins University School of Medicine, BaltimoreMD, United States; ^2^McKusick-Nathans Institute of Genetic Medicine, Johns Hopkins University School of Medicine, BaltimoreMD, United States

**Keywords:** ExAC, dilated cardiomyopathy, progeria, type 2 diabetes, FPLD2, metabolic syndrome, African American, Latino

## Abstract

Mutations in *LMNA*, encoding nuclear intermediate filament proteins lamins A and C, cause multiple diseases (‘laminopathies’) including muscular dystrophy, dilated cardiomyopathy, familial partial lipodystrophy (FPLD2), insulin resistance syndrome and progeria. To assess the prevalence of *LMNA* missense mutations (‘variants’) in a broad, ethnically diverse population, we compared missense alleles found among 60,706 unrelated individuals in the ExAC cohort to those identified in 1,404 individuals in the laminopathy database (UMD-LMNA). We identified 169 variants in the ExAC cohort, of which 37 (∼22%) are disease-associated including p.I299V (allele frequency 0.0402%), p.G602S (allele frequency 0.0262%) and p.R644C (allele frequency 0.124%), suggesting certain *LMNA* mutations are more common than previously recognized. Independent analysis of *LMNA* variants via the type 2 diabetes (T2D) Knowledge Portal showed that variant p.G602S associated significantly with type 2 diabetes (*p* = 0.02; odds ratio = 4.58), and was more frequent in African Americans (allele frequency 0.297%). The FPLD2-associated variant I299V was most prevalent in Latinos (allele frequency 0.347%). The ExAC cohort also revealed 132 novel *LMNA* missense variants including p.K108E (limited to individuals with psychiatric disease; predicted to perturb coil-1B), p.R397C and p.R427C (predicted to perturb filament biogenesis), p.G638R and p.N660D (predicted to perturb prelamin A processing), and numerous Ig-fold variants predicted to perturb phenotypically characteristic protein–protein interactions. Overall, this two-pronged strategy— mining a large database for missense variants in a single gene (*LMNA*), coupled to knowledge about the structure, biogenesis and functions of A-type lamins— revealed an unexpected number of *LMNA* variants, including novel variants predicted to perturb lamin assembly or function. Interestingly, this study also correlated novel variant p.K108E with psychiatric disease, identified known variant p.I299V as a potential risk factor for metabolic disease in Latinos, linked variant p.G602 with type 2 diabetes, and identified p.G602S as a predictor of diabetes risk in African Americans.

## Introduction

*LMNA* (MIM 150330) encodes two abundant proteins, lamins A and C, which are derived from alternative spliceforms of *LMNA* (Gruenbaum and Foisner, 2015) and form separate nuclear intermediate filaments important for nuclear structure, mechanical integrity and tissue-specific genome organization and signaling (Dauer and Worman, 2009; Burke and Stewart, 2014; Dialynas et al., 2015; Mattout et al., 2015; Osmanagic-Myers et al., 2015; Perovanovic et al., 2016; Le Dour et al., 2017). Mutations in *LMNA* cause at least eight autosomal dominant phenotypes and five autosomal recessive phenotypes, termed laminopathies (Bonne et al., 1999; Bertrand et al., 2011). These include Emery-Dreifuss Muscular Dystrophy (EDMD2, MIM 181350; EDMD3, MIM 616516), dilated cardiomyopathy (DCM; MIM 115200), congenital muscular dystrophy (CMD; MIM 613205), limb-girdle muscular dystrophy (LGMD; MIM 159001), Charcot-Marie-Tooth disease (CMT2B1; MIM 605588), arthropathy, tendinous calcinosis, and progeroid syndrome (ATCP; Van Esch et al., 2006), familial partial lipodystrophy type 2 (FPLD2; MIM 151660), insulin resistance syndrome (IRS; Young et al., 2005), mandibuloacral dysplasia type A (MADA; MIM 248370) and Hutchinson-Gilford Progeria Syndrome (HGPS; MIM 176670). The range of phenotypes in individuals with *LMNA* mutations is astonishing, from individuals who are wheelchair-bound, to phenotypically normal individuals or an Olympic athlete (Epstein, 2016). The underlying molecular mechanisms and tissue specificity are poorly understood.

*LMNA* missense mutations are also reported in patients with metabolic syndrome (Decaudain et al., 2007; Dutour et al., 2011), although genetic causality was not established. Affecting up to 30% of adults (Grundy, 2008), metabolic syndrome refers to combinations of traits that increase risk of type 2 diabetes, heart disease or stroke, each of which is influenced by multiple genes and environmental factors. The heterogeneity of metabolic phenotypes caused by *LMNA* mutations (Lewandowski et al., 2015; Chan et al., 2016) motivated us to assess the frequency of *LMNA* missense variants in broad populations and evaluate their potential to influence disease risk.

To explore *LMNA* variants in a relatively broad population, we analyzed *LMNA* missense alleles among the 60,706 unrelated individuals in the ExAC cohort (Lek et al., 2016). The ExAC cohort includes both genders (44.6% female) and is ethnically diverse (8.6% African/African-American; 9.5% Latino; 7.1% East Asian; 5.4% Finnish; 55% non-Finish European; 13.6% South Asian; 0.7% other). ExAC is devoid of individuals with severe pediatric-onset disorders and blends 14 cohorts including the Myocardial Infarction Genetics Consortium (14,622 individuals), Swedish Schizophrenia and Bipolar Studies (12,119 individuals), three Type 2 Diabetes consortia (GoT2D, SIGMA-T2D, T2D-GENES; 15,327 individuals), the Cancer Genome Atlas (TCGA; 7,601 individuals) and Inflammatory Bowel Disease (1,675 individuals), with similar numbers of patients and control individuals in each cohort. We compared the *LMNA* missense variants present in ExAC (Supplementary Table 1) to those found in 1,404 individuals in the UMD-LMNA database^1^. Certain variants in the UMD-LMNA database were excluded from our analysis, including the p.G608G variant responsible for progeria (‘progerin’; severe pediatric-onset disease) and all other non-missense mutations (deletions, insertions, synonymous, intronic, UTR, splice site, nonsense; 383 individuals). We defined novel variants as those found in ExAC that were not previously reported in the laminopathy database (compared in detail in Supplementary Table 2). Variants deemed novel are listed in Supplementary Table 3, along with previously reported SNP identifiers for specific variants. Novel variants were evaluated in the context of the lamin polypeptide to predict their potential impact on lamin structure or function.

## Materials and Methods

### Accession of *LMNA* Variants Reported in ExAC

Version 0.3.1 ExAC data was accessed by querying “LMNA” through the ExAC Browser (Beta) (ExAC database, 2017). All data were reported as PASS per ExAC data quality standards. Data was selected for “Missense + LoF” variants and exported to CSV. The data was further narrowed to include only missense variants of canonical spliceforms of lamins A and C (Supplementary Table 1).

### Accession of *LMNA* Mutations Reported in the UMD-LMNA Database

The full *LMNA* list of mutations (311 references and 2251 subjects) was accessed from the UMD-LMNA database [UMD-LMNA (laminopathy) database, 2017]. This list was then curated to exclude all non-missense mutations, and non-canonical spliceforms, yielding the 1404 variants shown in Supplementary Table 2 (comparison of ExAC and UMD-LMNA variant data).

### Independent Analysis of *LMNA* Variants in the T2D Knowledge Portal

Since we were unable to determine disease status for specific variants in the ExAC cohort, we accessed and searched *LMNA* variants in the Type 2 Diabetes Knowledge Portal^2^ using a Type 2 Diabetes phenotype filter. Search results were selected for variants of “nominal significance,” defined by the T2D Knowledge database as having a *p*-value < 0.05. Variants significant for Type 2 Diabetes were identified solely within the 17K exome sequence analysis cohort, which comprises data from T2D-GENES (1,018 case and 1,056 control African Americans; 1,012 case and 1,153 control East Asians; 990 case and 853 control Europeans; 1,021 case and 922 control Latinos; 1,094 case and 1,123 control South Asians), GoT2D (1,369 case and 1,339 control Europeans) and SIGMA (1,817 case and 1,975 control Latinos). Detailed information about these studies can be found at http://www.type2diabetesgenetics.org/informational/data. The link between variant p.G602S and type 2 diabetes was found in three subcohorts within the T2D-GENES dataset, namely the Jackson Heart Study Candidate Gene Association Resource and the Wake Forest Study (both enriched in African American individuals), and the Singapore Indian Eye Study (enriched in South Asian individuals). All p.G602S-related statistics were obtained from the analysis reported in the T2D Knowledge Portal. This independent analysis of the T2D-GENES dataset, via the T2D Knowledge Portal, was essential to associate variant p.G602S with diabetes (even though these individuals are included in the ExAC cohort), since patient data in the T2D-GENES cohort was not accessible via ExAC.

### Selection of Data for Psychiatric Cohort

ExAC release 0.3 has two variant call format (.vcf) files: ExAC.r0.3.sites.vep.vcf.gz, which contains the variant calls for all 60,706 individuals, and ExAC.r0.3.nonpsych.sites.vcf.gz, which contains the variant calls from 45,376 individuals from the non-psychiatric subset. To determine if any variants were enriched in the psychiatric cohort, we identified *LMNA* missense alleles unique to the remaining 15,330 individuals from the psychiatric subset, comprised of the Swedish Schizophrenia and Bipolar Studies cohort (12,119 individuals), Schizophrenia Trios from Taiwan (1,505 individuals), Sequencing in Suomi (948 individuals), Bulgarian Trios (461 individuals) and Tourette Syndrome Association International Consortium for Genomics (297 individuals). We used 7zip to extract the.vcf files. In a Cygwin64 terminal, header information was removed and only the chromosome number, position, reference sequence, and alternate sequence fields were retained. We generated a psychiatric-unique file by retaining lines from the “all sites” variant call file that were not present in the non-psychiatric subset file. The consequences of multi-allelic or minimal representation were handled manually in vim to ensure that all entries in the psychiatric-unique.vcf file were not also present in the non-psychiatric subset.

**Shell script used to select data from psychiatric cohorts within ExAC:**

#*!/bin/tcsh*

# *Shell Script to Generate.vcf Files with Psychiatric-Unique Alleles*

# *remove header information*

grep -v ′ˆ#′ ExAC.r0.3.sites.vep.vcf > allsites_cln.vcf

grep -v ′ˆ#′ ExAC.r0.3.nonpsych.sites.vcf > nonpsych_cln.vcf

# *retain only chromosome no., position, refseq, and altseq fields*

awk ′{$3 = $6 = $7 = $8″″; print $0}′ allsites_cln.vcf > allsites_final.vcf

awk ′{$3 = $6 = $7 = $8″″; print $0}′ nonpsych_cln.vcf > nonpsych_final.vcf

# *subtract nonpsych subset from allsites*

comm -23 allsites_final.vcf nonpsych_final.vcf > psych_final.vcf

# *create file for just chromosome 1*

awk ′$1== 1′ nonpsych_final.vcf > chromo1_nonpsych.vcf

awk ′$1== 1′ psych_final.vcf > chromo1_psych.vcf

# *select all positions corresponding to LMNA*

awk ′$2>=156082546 && $2<=156140089′ chromo1_nonpsych.vcf>LMNA_nonpsych.vcf

awk ′$2>=156082546 && $2<= 156140089′ chromo1_psych.vcf>LMNA_psych.vcf

### Sequence Alignment of Modular Domains

The amino acid sequence alignments shown in **Figures 3, 5** were performed manually, using Accession identifiers provided in figure legends. The human lamin A, B1 and B2 sequences used in the alignment of head and ‘neck’ domains (**Figures 3A,D**) were accessed through UniProtKB. We also used UniProtKB to access lamin A sequences from *Camelus ferus, Dasypus novemcinctus*, and *Danio rerio* for alignment with human lamin A (**Figure 5A**).

### Predictive Modeling of Coiled-Coil Interactions between Rod Domains

Native coiled-coil interactions between two parallel rod domains of A-type lamins were predicted using DrawCoil 1.0^3^. Comparative predictions were made with single missense variant sequences, as described in **Figures 3B,C** legends.

## Results

**FIGURE 1:**
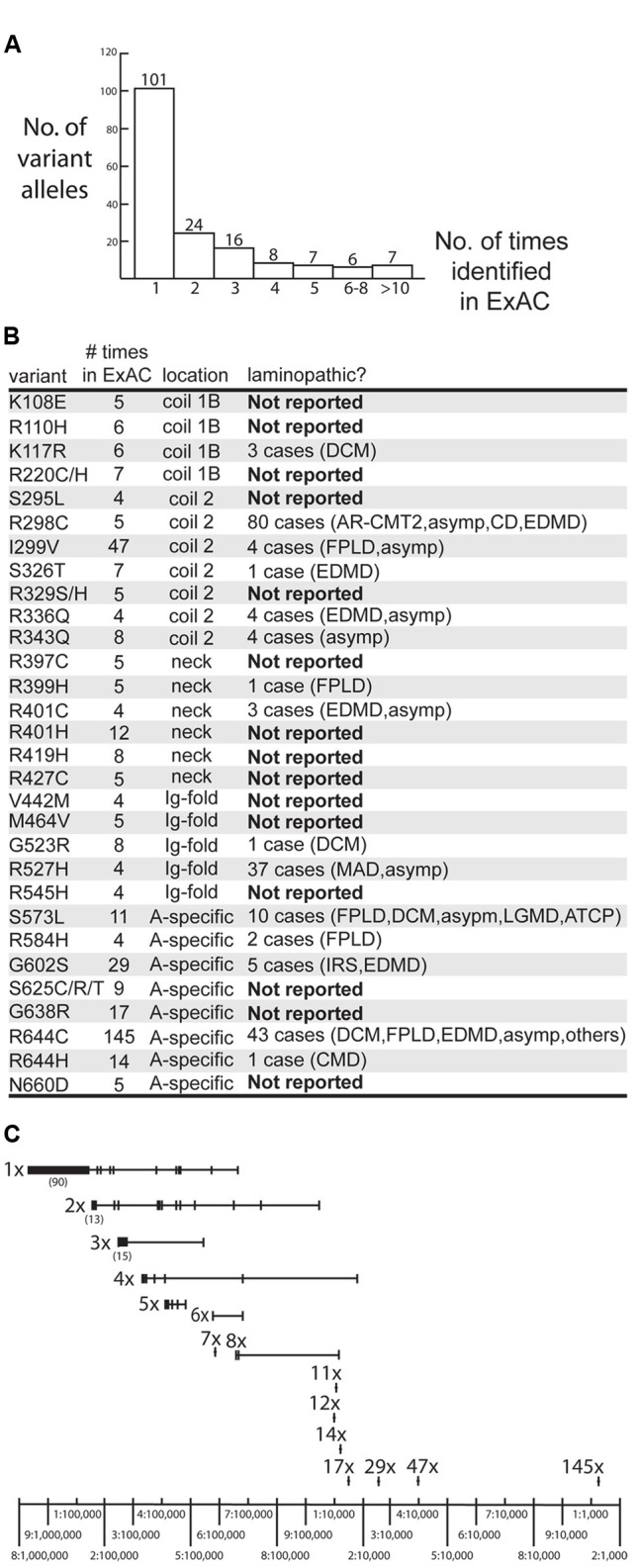
Overview of *LMNA* missense variants identified in the ExAC cohort. **(A)** Graph depicting the number of times each variant allele was identified in the ExAC cohort. Among the 169 identified variants, 101 were each identified in a single individual. **(B)** Information about specific variants identified four or more times in the ExAC cohort, including the number of times each variant was identified in ExAC, its molecular location in the lamin polypeptide (‘location’), and whether it is novel (‘Not Reported’), defined as not reported in the laminopathy database (comprising patients and relatives *with the same *LMNA* mutation). *AR-CMT2*, autosomal recessive Charcot-Marie-tooth disease type 2. *Asymp*, asymptomatic. *ATCP*, arthropathy, tendinous calcinosis, and progeroid syndrome. *CD*, cardiac disease. *CMD*, congenital muscular dystrophy. *DCM*, dilated cardiomyopathy. *EDMD*, Emery-Dreifuss muscular dystrophy. *FPLD2*, familial partial lipodystrophy type 2. *IRS*, insulin resistance syndrome. *LGMD*, limb-girdle muscular dystrophy. *MADA*, mandibuloacral dysplasia type A. **(C)** Schematic depicting the frequency of variant alleles (denoted by short vertical lines). Alleles are grouped on the same horizontal line, based on whether they were identified in a single individual (‘1x’), two individuals (‘2x’) and so on. Most variants were rare (∼1–5 per 100,000 sequenced chromosomes) in the ExAC population. Relatively frequent variants included p.G638R (‘17x’), p.G602S (‘29x’) and p.R644H (‘47x’; ∼1–4 per 10,000 sequenced chromosomes) and p.R644C (‘145x’; >1 per 1,000 sequenced chromosomes).*

The ExAC database included 169 missense alleles (‘variants’) of prelamin A (reference sequence GRCh37/hg19; Supplementary Table 1). *LMNA* had an ExAC loss intolerance (pLI) score of 0.99, meaning that *LMNA* is highly conserved with fewer loss-of-function variants and lower frequencies for observed variants than typical protein-coding genes. Genes with pLI scores >0.90 are considered loss-intolerant (e.g., encoding proteins with multiple binding partners, or haploinsufficient proteins in which heterozygous alleles are likely to cause dominant disorders; Lek et al., 2016), consistent with lamins as structural polymers with diverse partners and the dominance of heterozygous mutations in *LMNA* (Simon and Wilson, 2013). *LMNA* had an ExAC missense Z score of 3.37, where positive *Z* scores indicate proteins that are intolerant of single residue variation (Lek et al., 2016). Most variants were identified in only one individual (101/169 or 60% of the *LMNA* variant alleles), two individuals (24/169 variant alleles; 14%) or three individuals (16/169 variant alleles; 10%; **Figure 1A**). Other variants were identified in 4–8 individuals (21/169 variant alleles; 12%) or 11–144 individuals (7/169 variant alleles; 4%; **Figure 1A**). The ExAC cohort revealed many novel *LMNA* variants, 15 of which were present in four or more (up to 17) individuals. Three of the most frequent *LMNA* variants in ExAC were novel: p.R401H (12 individuals), p.R419H (8 individuals) and p.G638R (17 individuals; **Figure 1B**). Other novel variants included p.K108E, p.R110H, p.R220C/H, p.S295L, p.R329S/H, p.R397C, p.R427C, p.V442M, p.M464V, p.R545H, p.S625C/R/T and p.N660D (**Figure 1B**; note p.R545H is now reported in one case of FPLD; Chan et al., 2016).

All *LMNA* variants in the ExAC cohort were considered rare (minor allele frequency <1%). Nevertheless, the frequencies of *LMNA* variant alleles in the ExAC database spanned two orders of magnitude, from roughly one-in-100,000 to one-in-1,000 individuals (**Figure 1C**).

### Evaluation of ExAC Variants Also Reported in the Laminopathy Database

**FIGURE 2:**
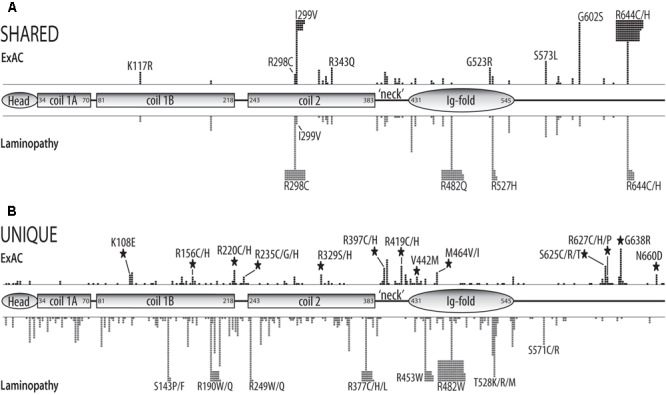
Schematic of structural and functional domains in the human prelamin A polypeptide (head, coiled-coil rod, ‘neck’, Ig-fold, unstructured C-terminus) showing the positions and numbers of missense variants in the ExAC vs. laminopathy cohorts. Each black square represents one individual in the ExAC cohort. Each gray square represents one individual in the laminopathy cohort. Individuals with different substitutions at the same position (e.g., p.R644C and p.R644H) are pooled. **(A)** Positions and numbers of ExAC variants also reported in the laminopathy cohort (‘shared’). **(B)** Positions and numbers of variants unique to the ExAC cohort or unique to the laminopathy cohort. Large black stars indicate that four or more individuals in the ExAC cohort have a variant at a ‘new’ residue; i.e., the laminopathy cohort lacks variants at this position.

We compared the missense variants reported in ExAC to those identified in 1,404 distinct individuals with missense mutations in the laminopathy database (Supplementary Table 2). Among the 28 variants identified four or more times in ExAC, 15 were also represented in the laminopathy database. Note that this fact alone does not predict overt disease, since 214 of the 1,404 individuals in the laminopathy database with missense mutations were reported as asymptomatic. However, only a minority of laminopathy-associated variants are reported in asymptomatic individuals. One variant (p.R343Q, found in eight ExAC individuals) is reported in a single apparently healthy individual in the laminopathy database. By contrast, other ‘shared’ variants were nearly always reported in individuals with disease in the laminopathy database (**Figure 1B** and Supplementary Table 2). These included p.G523R (8 individuals in ExAC; one DCM patient in laminopathy cohort), p.S573L (11 individuals in ExAC; 10 individuals with multiple phenotypes in laminopathy cohort), p.R644H (14 individuals in ExAC; one patient with congenital muscular dystrophy in laminopathy cohort), p.G602S (29 individuals in ExAC; 3 patients with IRS and 2 patients with EDMD in laminopathy cohort), p.I299V (47 individuals in ExAC; one asymptomatic individual and 3 FPLD2 patients in laminopathy cohort), and p.R644C (145 alleles corresponding to 144 individuals in ExAC; 43 patients with multiple phenotypes including DCM and FPLD2 in laminopathy cohort) (**Figure 1B**). All variants were observed in heterozygosity in both the ExAC and laminopathy databases, with rare exceptions as noted. All variants in the ExAC cohort and laminopathy cohort and their locations in the prelamin A polypeptide are depicted in **Figure 2**. Variants found in both cohorts are shown in **Figure 2A**; variants unique to each cohort are shown in **Figure 2B**. More than 219 individuals with *LMNA* variants in the ExAC cohort have a mutation previously observed in patients with laminopathies— predominantly but not exclusively heart disease— including 158 individuals with either p.R644C or p.R644H, and 61 individuals with p.K117R, p.S326T, p.G523R, p.S573L, or p.G602S (**Figure 2A**). Because individual-level phenotypic data is unavailable for the ExAC cohort, we were unable to determine whether these variants correlated with myocardial disease or other specific phenotypes. However, since there is already strong evidence that *LMNA* mutations contribute to heart disease in the general population (Parks et al., 2008; Tesson et al., 2014), these variants are of particular interest for functional follow-up.

There were 145 p.R644C alleles in the ExAC cohort, representing 144 individuals (one homozygote), out of 116,680 total alleles for an allele frequency of 0.1242%. For phenotypic perspective, the laminopathy database reports 43 individuals with p.R644C, among whom 11 were asymptomatic, one had atypical progeria, one had neuropathy, six had lipodystrophy, and 23 had cardiac disease with or without muscular dystrophy. Although these discrepant phenotypes make it difficult to ascertain the true consequences or pathogenicity of this allele, these results warrant further study of the ExAC cohort to determine what percentage of individuals with p.R644C are asymptomatic and whether this variant correlates with any diseases in this population. A different variant at this same amino acid residue, p.R644H, is seen once in heterozygosity in the laminopathy cohort (a girl with congenital muscular dystrophy who died at 20 months). We note that p.R644H was identified in heterozygosity in 14 of 116,722 chromosomes in the ExAC cohort, for an allele frequency of 0.0120%. Because all individuals represented in ExAC survived childhood, the pathogenicity of this variant in isolation is dubious. Interestingly, p.R644C is ethnically clustered within ExAC. This variant is more frequent in non-Finnish Europeans (106 out of 63,724 sequenced alleles; 0.162% allele frequency) and South Asians (27 out of 16,410 sequenced alleles; 0.165% allele frequency). No obvious ethnic correlation was found for p.R644H.

The *LMNA* p.I299V variant was identified 47 times in the ExAC cohort (heterozygous individuals; allele frequency 0.0402%). Four related individuals in the laminopathy database have this variant: a heterozygous mother and two heterozygous daughters all with FPLD2, and a grandson homozygous for p.I299V who presented as asymptomatic (Araújo-Vilar et al., 2012). This heterogeneity is consistent with FPLD2 as a progressive and often misdiagnosed disorder with variable phenotypic manifestations and age of onset (MIM 151660; Hussain and Garg, 2016). Individuals harboring this variant in ExAC might have features of FPLD2. Alternatively, this variant may not be solely responsible for FPLD2. We conclude that p.I299V, the second most common variant in the ExAC cohort, has the potential to correlate with disease risk and warrants further genotype–phenotype analysis. Upon analyzing the ethnic sub-cohorts of ExAC, we found that p.I299V is more frequent among Latino individuals (39 of the 11,242 alleles; 0.347% allele frequency). The concentration of variant p.I299V among Latinos warrants further investigation as a potential predictor of disease risk.

One individual in the ExAC cohort was heterozygous for the *LMNA* variant p.R482Q. Variants p.R482Q and p.R482W are considered rare and highly penetrant mutations responsible for FPLD2, and are seen in ∼13% of all known laminopathy patients (241 of 1849 total individuals in the laminopathy database). p.R482 substitutions are implicated in 80% of FPLD2 cases, which has a disease prevalence of less than 1 in 10 million (Subramanyam et al., 2010). It is possible that the individual in ExAC who carries p.R482Q has unrecognized features of FPLD2. Alternatively, the variant might have been non-penetrant in this individual at the time of study. Further investigation in databases that provide individual genotype/phenotype data will be crucial to understanding the implications of this variant in the broader population.

### Variant p.G602S Associates with Type 2 Diabetes, and Is More Frequent in African Americans

The p.G602S variant was identified 29 times in heterozygosity in the ExAC cohort with an overall allele frequency of 0.0262%. The laminopathy database reports only five individuals with p.G602S: two males (relation uncertain) with EDMD (Bakay et al., 2006; Scharner et al., 2011) and three related individuals from Reunion Island (African Creole descent), including a father with Type 2 diabetes, his son with insulin-resistant diabetes, and daughter with polycystic ovarian syndrome and insulin resistance (Young et al., 2005). To explore the implications of the ExAC data, we analyzed the open-access Type 2 Diabetes Knowledge Portal (Type 2 Diabetes Knowledge Portal, 2016) and searched within 100 kb of *LMNA*. This search revealed 118 variants in the 17K exome sequence analysis dataset, but only one variant showed a significant correlation with disease: p.G602S was identified in 14 out of 8378 patients with Type 2 diabetes (0.17% of patients), and in 2 of 8478 controls (0.024% of controls; odds ratio 4.58; *p*-value 0.0200). Interestingly, 13 of the 14 individuals with Type 2 Diabetes in the 17K Exome cohort were African American and one was South Asian. This proposed link to Type 2 Diabetes is consistent with the above-discussed family in the laminopathy database in which the p.G602S variant co-segregates with certain aspects of diabetes in two generations of a family with African ancestry (Young et al., 2005). Analysis of the ExAC database using ethnic break-out information shows that 26 of the 29 individuals with p.G602S were African American; by contrast the European American, Latin American, and South Asian cohorts each included a single individual with p.G602S. The allele frequency for p.G602S within the African American cohort in ExAC was 0.297% (26 out of 8,766 total alleles). These results warrant further studies of the effects of the *LMNA* p.G602S variant on glucose homeostasis, and further consideration as a contributor to diabetic risk, particularly in individuals with African ancestry.

### Potential Enrichment of Variant p.K108E in Individuals with Psychiatric Disease

To determine whether any *LMNA* missense variants were enriched in individuals with psychiatric disease, we extracted variant call files from the subset of individuals in ExAC with recognized psychiatric disorders (see Materials and Methods). There are several limitations to this approach. For instance, we could not be certain that all control individuals were excluded, and individuals with psychiatric disease might also have concurrent clinical phenotypes (e.g., heart disease) attributable to *LMNA* variants. In addition, we could not determine whether individuals in the psychiatric cohort with the same mutation had the same psychiatric phenotype (e.g., schizophrenia vs. bipolar disorder vs. Tourette). Finally, it is possible that certain individuals in the non-psychiatric subset had undiagnosed psychiatric conditions.

With these caveats in mind, 19 *LMNA* variants were present only in the psychiatric cohort in ExAC. The allele frequencies of these variants among all 60,706 individuals represented in ExAC ranged from <0.001 to 0.004829%. Four such variants, identified once each, are also reported in the laminopathy database: p.S395L (one individual with IRS; Decaudain et al., 2007), p.V586M (one individual with striated muscle laminopathy; Rodriguez et al., 2008), p.R388H (two DCM patients and 5 asymptomatic individuals; Parks et al., 2008) and p.S583L (9 individuals with FPLD; Savage et al., 2004). The remaining 15 variants were all novel (Supplementary Table 3). Since most were identified in only one individual, or two individuals each for variants p.Q168H, p.R235H, and p.Q396L, their potential significance is unknown. Intriguingly, all five individuals with variant p.K108E (overall allele frequency 0.004829% in ExAC) were unique to the psychiatric cohort and were all non-Finnish Europeans. This variant has potential molecular consequences, as discussed below.

### Potential Molecular Impacts of Novel Variants on Lamin Polypeptides

Lamins A and C have identical residues 1–566, but have different C-terminal sequences generated by alternative splicing (Luo et al., 2014). Both isoforms are widely expressed throughout most human tissues except brain, which selectively expresses lamin C (Jung et al., 2012). Lamin C has six additional unique C-terminal residues, whereas lamin A is synthesized as a precursor (prelamin A) containing additional residues 567–664. Prelamin A is post-translationally modified and finally cleaved by the protease ZMPSTE24 to generate mature lamin A (comprising residues 1–644; Sinensky et al., 1994; Wang et al., 2016). Missense mutations linked to known laminopathies are generally scattered throughout the polypeptide and can alter many aspects of lamin biology, from filament assembly to partner interactions and post-translational modifications (Simon and Wilson, 2013). This functional complexity poses challenges to understanding disease mechanisms, and motivated our search for new human *LMNA* variants that might provide molecular insights.

**FIGURE 3:**
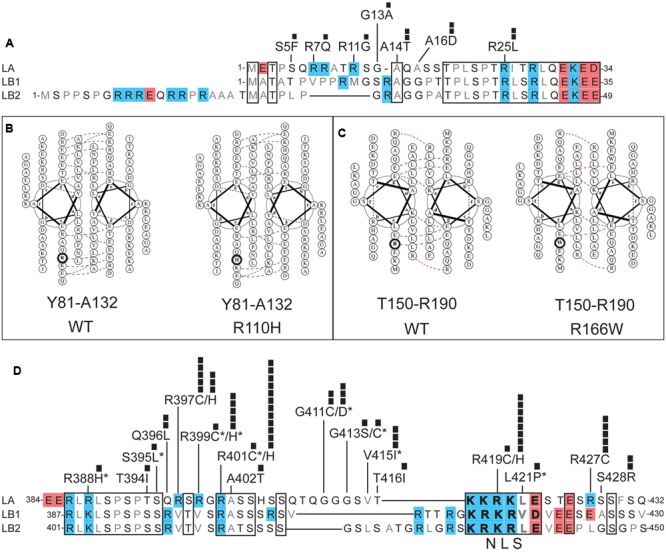
Positions and potential consequences of novel variants in the head, coiled-coil rod or neck domains. **(A)** Head domain amino acid sequences of human lamin A (LA; UniProt P02545), lamin B1 (LB1; UniProt P20700) and lamin B2 (LB2; UniProt Q03252). Regions conserved among LA, LB1, and LB2 are boxed. Polar residues are lettered in black; hydrophobic residues in gray. Each black square represents one individual with the indicated variant. **(B,C)** Loss of coiled-coil backbone associations between parallel rod domains predicted for: **(B)** novel variant p.R110H in the context of wild-type (‘WT’) residues Y81-A132, and **(C)** novel variant p.R166W in the context of wild-type residues T150-R190. Dotted lines indicate ionic interactions between two residues, as predicted by DrawCoil 1.0 (http://www.grigoryanlab.org/drawcoil/). **(D)** Amino acid sequences of the neck domains of human lamin A (residues 384–432), lamin B1 (residues 387–430) and lamin B2 (residues 401–450). Nuclear localization signal (NLS) residues are bolded. Specific ExAC variants, and the number of individuals (black squares) with each variant, are shown. Asterisks indicate ExAC variants also reported in the laminopathy database.

**FIGURE 4:**
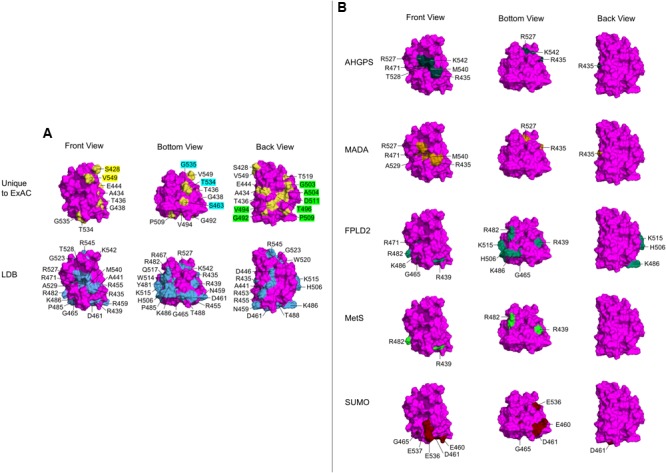
Ig-fold surface residues and ‘features’ affected by ExAC vs. laminopathy variants. Each row shows three views of the Ig-fold, determined by NMR (RCSB Protein Data Bank ID: 1IVT). Front Views depict R542 facing out of the page, Bottom Views depict R542 rotated 90° up and slightly to the right, and Back Views depict R542 facing into the page. (Residue L530 is surface exposed, but not visible in any view). Wild-type residues affected by variants are labeled, and shaded yellow in the atomic structure. **(A)** The upper row shows wild-type residues affected by variants unique to the ExAC cohort (‘Unique to ExAC’). The bottom row shows all surface residues affected by reported missense variants in the laminopathy database (‘LDB’). Residues that form molecular ‘features’ uniquely affected by ExAC variants are indicated in the Front view (yellow labels), Bottom view (blue labels) and Back view (green labels). **(B)** Selective depiction of residues impacted by variants in the laminopathy database that cause either atypical HGPS (‘AHGPS’), the progeroid-spectrum disorder MADA or FPLD2, and variants reported in patients with metabolic syndrome (‘MetS’) (Decaudain et al., 2007; Dutour et al., 2011). The bottom row depicts FPLD2-related residues required for SUMO1 modification of Ig-fold residue K486 (‘SUMO’; Simon et al., 2013).

**FIGURE 5:**
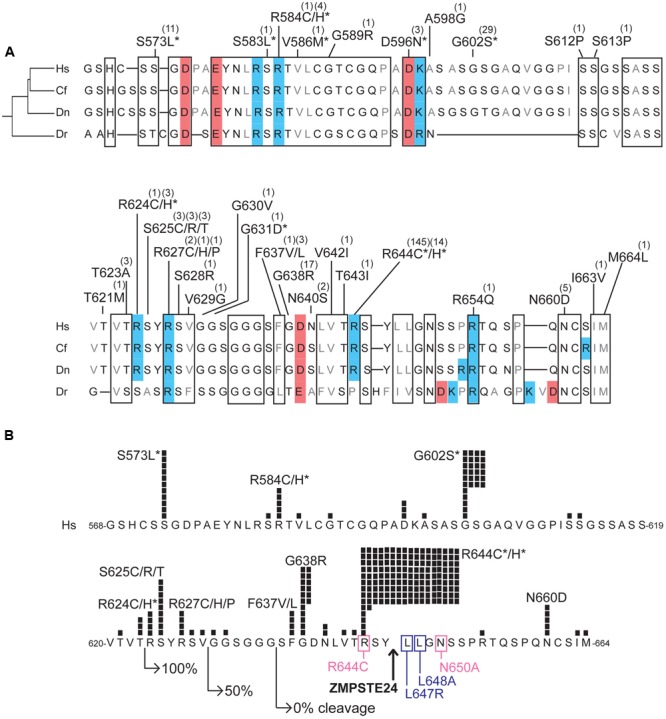
Variants located in the unstructured C-terminal region unique to prelamin A. **(A)** Aligned amino acid sequences of the C-terminal region of prelamin A (human residues 568–664) in *Homo sapiens* (‘Hs’; UniProt P02545), *Camelus ferus* (‘Cf’; camel; Refseq XP_014422471.1), *Dasypus novemcinctus* (‘Dn’; nine-banded armadillo; Refseq XP_004462616.1), and *Danio rerio* (‘Dr’; zebrafish; GenBank AAI63807.1). These animals were selected to highlight differences among *LMNA* residues in a diverse array of species; residues conserved among these species, aligned manually and using Clustal Omega (http://www.ebi.ac.uk/Tools/msa/clustalo/) are likely to be functionally important. Highly conserved regions are boxed. Acidic residues are shaded red; basic residues are shaded blue. ExAC variants are indicated; parentheses show the number of affected individuals. Asterisks indicate variants also reported in the laminopathy database. **(B)** ExAC variants and number of affected individuals (black squares) in prelamin A residues 568–664, in the context of regions and residues important for ZMPSTE24-dependent cleavage. Right-pointing arrows indicate the N-terminal residue of fragments tested for ZMPSTE24-dependent cleavage in mammalian cells; GFP-fused fragments comprising residues 624–664 were cleaved efficiently (100%), residues 630–664 were cleaved inefficiently (50% cleaved) and residues 636-664 remained uncleaved (Barrowman et al., 2012). Upward arrow: ZMPSTE24 cleavage site. Missense mutations that reduce cleavage efficiency (pink box) or abrogated cleavage (purple box; Barrowman et al., 2012) are indicated. Asterisks indicate variants also reported in the laminopathy database.

We searched for novel missense variants in lamin C-specific residues, but found none. We therefore considered all missense variants in the context of their molecular locations in the prelamin A polypeptide, which includes all but six residues of lamin C. Different regions of the prelamin A protein, diagramed in **Figure 2**, are considered in detail below. The globular N-terminal ‘head’ domain (residues 1–34; **Figure 3A**), long coiled-coil ‘rod’ domain (residues 35–383; **Figures 3B,C**), small flexible ‘neck’ domain (residues 384–430; **Figure 3D**) and Ig-fold domain (residues 431–545; **Figure 4**) are all identical in lamins A and C. The unstructured C-terminal region unique to prelamin A (residues 568–664) that is processed to generate mature lamin A (residues 568–646) is depicted in **Figure 5**.

### Head Variants That Might Affect Head-to-Tail Polymerization

The head domain of human lamin A is 47% identical in lamins B1 and B2 (**Figure 3A**). This conservation is consistent with evidence that lamin A head residues 11–30 are required for head-to-tail polymerization *in vitro* (Isobe et al., 2007). Residues R7, R11, and R25 are predicted to mediate electrostatic interactions with coil-2B (Strelkov et al., 2004), suggesting head-to-tail polymerization may be weakened by novel ExAC variants p.R7Q, p.R11G and p.R25L (**Figure 3A**). Post-translational regulation or other functions of the head domain might be perturbed by novel ExAC variants p.S5F (Ser5 is a conserved phosphorylation site; Santamaria et al., 2011; Kochin et al., 2014), and by non-conservative substitutions such as p.A14T and p.A16D (**Figure 3A**). Head domain variants were each identified in only 1–2 individuals (**Figure 3A**).

### Coiled-Coil ‘Rod’ Domain Variants

The coiled-coil ‘rod’ domain is a defining structural feature of intermediate filament proteins (Herrmann and Aebi, 2016). In lamins, the rod domain comprises three regions (coil-1A, coil-1B, and coil-2) separated by short linkers. The rod domains of two nascent lamin polypeptides associate in parallel, creating a structural coiled-coil motif (Heitlinger et al., 1992). We used DrawCoil 1.0 (developed in the Grigoryan lab at Dartmouth University) to predict the consequences of variants in the rod domain. Based on the predicted loss of conserved electrostatic ‘backbone’ interactions between rod domains, we suggest that lamin dimerization would be perturbed by novel variants p.R110H (identified in 6 individuals; allele frequency 0.00483%; **Figure 3B**) and p.R166W (two individuals; allele frequency 0.00384%; **Figure 3C**). Different amino acid substitutions at these same positions are seen in the laminopathy database: p.R110S (one DCM patient and 3 asymptomatic individuals; Botto et al., 2011) and p.R166P (3 patients with DCM; Parks et al., 2008; Saga et al., 2009). In addition to structural roles, the rod domain is post-translationally regulated and provides functional docking sites for specific partners (e.g., retinoblastoma protein binds lamin A/C residues 247–355; Mancini et al., 1994; Ozaki et al., 1994). Interestingly, novel variant p.K108E (5 individuals in the psychiatric cohort; discussed above) affects the rod domain surface, and would block two known post-translational modifications at K108: acetylation and ubiquitylation (Simon and Wilson, 2013).

### ‘Neck’ Variants That Might Affect Head-to-Tail Polymerization or Other Roles

Head-to-tail polymerization is further predicted to involve four Arg residues (R388, R397, R399, and R401) in the ‘neck’ region, which contact coil-1A in the partner (Strelkov et al., 2004). These interactions may be altered by ExAC variants p.R388H (one individual), p.R397C (novel; 5 individuals; allele frequency 0.004176%), p.R397H (novel; 3 individuals; allele frequency 0.002505%), p.R399C (one individual), p.R399H (5 individuals; allele frequency 0.004169%), and p.R401H (novel; 12 individuals; allele frequency ∼0.01%; **Figure 3D**). The positively charged nuclear localization signal (NLS; residues 417–422; **Figure 3D**) in the neck region is likely to be perturbed by novel variant p.R419C (one individual, allele frequency 0.0008268%), and possibly by novel variant p.R419H (8 individuals; allele frequency 0.006629%; **Figure 3D**). Variant p.R419C was reported in three FPLD patients, although pathogenicity was not clearly established (Haque et al., 2003). Two studies in cells with exogenously expressed p.R419C reached conflicting conclusions about its effects on nuclear localization (Yang et al., 2013; Kiel et al., 2014). Novel variants p.G411C (2 individuals; allele frequency 0.001660%), p.R427C (5 individuals; allele frequency 0.00416%), and p.S428R (2 individuals; allele frequency 0.001670%) are non-conservative substitutions that might intrinsically perturb neck flexibility or function (**Figure 3D**). For example, neck residues 403–425 mediate binding to nesprin-2, a component of nuclear envelope-spanning ‘LINC’ complexes (Yang et al., 2013). The ExAC cohort included single individuals with neck variants also reported in individuals with disease in the laminopathy database: p.G411D (IRS; Dutour et al., 2011), p.G413C (striated muscle disease; Rodriguez et al., 2008) and p.V415I (isolated atrial fibrillation; Brauch et al., 2009).

### Novel Variants Affect Specific Molecular ‘Features’ on the Ig-Fold Surface

Novel ExAC variants in the structurally conserved Ig-fold domain were identified in single individuals (19 variants) or in two (p.S428R), three (p.A434T, p.R453Q), four (p.V442M, p.A545H) or five (p.M464V) individuals (**Figure 2B** and Supplementary Table 2). The ConSurf algorithm (ConSurf database, 2017), which aligns solved protein structures with all known structural homologs to assess evolutionary conservation, showed that six evolutionarily invariant residues were affected by ExAC variants: three are known to associate with disease (p.D461Y, p.R471C, and p.G523R); the others are novel non-conservative substitutions (p.S428R, p.T436I, and p.G438R) that are likely to perturb Ig-fold function.

Two slightly different atomic structures are reported for the Ig-fold domain of human lamin A. One was determined by X-ray crystallography (RCSB Protein Data Bank ID: 1IFR) for residues 432–544, but includes non-native N-terminal residues 432–434 (GSH) and a glycerol ligand (Dhe-Paganon et al., 2002). This ligand is predicted by PyMOL to form polar contacts with a backbone hydroxyl group on V513. The other structure, determined in solution by NMR (RCSB Protein Data Bank ID: 1IVT) is longer (residues 428–549), has native residues 432–434 (QHA), and no ligands (Krimm et al., 2002). This NMR structure consists of 15 frames; we used the first frame, which is representative as seen in the superimposition^4^. We used PyMOL to map the positions of wild-type residues corresponding to each novel variant (‘Unique to ExAC’; **Figure 4A**). We compared these to all surface-exposed residues affected by variants in the laminopathy database (‘LDB’; **Figure 4A**). We obtained similar results for both structures, but show only the NMR results because this structure includes affected residues S428, A434, R545, and V549.

With one exception (p.L530V), all Ig-fold variants unique to the ExAC cohort affect wild-type residues that are solvent-accessible (‘Unique to ExAC’; **Figure 4A**; note that M464, in a tiny cavity slightly behind P509, is not visible). By contrast, most variants linked to EDMD affect residues in the hydrophobic interior, or residues required to stabilize Ig-fold structure (e.g., R453; Burke and Stewart, 2002; Scharner et al., 2014). Surface-exposed residues linked to skeletal muscle disease include D446, R453, R455, N459, R545, and W520, roughly aligned on one side of the Ig-fold (Scharner et al., 2014), as seen in our ‘Back’ view (‘LDB’; **Figure 4A**). Three residues in this ‘muscle-region’ were affected in the ExAC cohort, but with substitutions more conservative (p.D446E, p.R453N, p.R545H in ExAC) than those linked to skeletal muscle disease (p.D446V, p.R453P, p.R545C; Supplementary Table 2).

Interestingly, a comparison of residues uniquely affected by ExAC variants (‘Unique to ExAC’; **Figure 4A**), compared to residues affected by all laminopathy variants (‘LDB’; **Figure 4A**), revealed that certain novel ExAC variants affect ‘new’ regions of the Ig-fold surface. In particular, ExAC variants at the evolutionarily invariant residue S428, and residue V549, affect two ‘knobs’ in the Front view (yellow labels; ‘Unique to ExAC’; **Figure 4A**). Different ‘knobs’ in the Bottom view are affected by ExAC variants at S463, T534, and G535 (aqua labels; **Figure 4A**). Seven novel ExAC variants affect residues G492, V494, T496, G503, A504, P509, and D511, best seen in the Back view (green labels; **Figure 4A**), adjacent to but distinct from the ‘muscle’ region (Scharner et al., 2014).

These proposed Ig-fold surface ‘features’ affected by novel ExAC variants were also distinct from three other previously recognized features. SUMO1 modification of Ig-fold residue K486, potentially relevant to FPLD2, requires an acidic ‘patch’ formed by E460, D461, E536, and E537 on the Ig-fold surface (‘SUMO’; **Figure 4B**; Simon et al., 2013). A different region of the Ig-fold surface is specifically affected by variants linked to progeria. In particular, atypical HGPS (AHGPS) and MADA, which have overlapping clinical phenotypes (Guénantin et al., 2014; Mehrez and Mostafa, 2014; Krawiec et al., 2016), are caused by single substitutions at residues R435, R471, R527, A529, M540, or K542, which form a ‘patch’ on the Ig-fold surface (Front views; AHGPS and MADA; **Figure 4B**). We exclude neighboring residue T528 as a core component of this ‘progeria patch’ because most T528 variants cause muscular dystrophy (Vytopil et al., 2003; Verstraeten et al., 2006; Astejada et al., 2007; Bertrand et al., 2011; Scharner et al., 2011). We consider residues A529, M540, and K542 as core features of the progeria patch, since variants p.A529V, p.A529T, and p.K542N each cause progeroid phenotypes in homozygosity (Plasilova et al., 2004; Garg et al., 2005; Kosho et al., 2007) and progeria also arises from compound heterozygosity for p.M540T and p.T528M (Verstraeten et al., 2006). No variants at these residues were seen in ExAC. However, the ExAC cohort included variants at three other progeria-relevant residues: R435, R471, and R527. One ExAC individual carried p.R435C (allele frequency 0.000863%); this variant is reported as heterozygous in 14 asymptomatic individuals in the laminopathy cohort and one DCM patient (Vytopil et al., 2003), and homozygously in single cases of progeria (Madej-Pilarczyk et al., 2009) or restrictive dermopathy (Youn et al., 2010). Three individuals in ExAC carried p.R471C (allele frequency 0.00256%); this variant is also reported in the laminopathy cohort in three asymptomatic heterozygous individuals, one homozygous patient with MADA/progeria (Zirn et al., 2008) and one HGPS patient in compound heterozygosity with p.R527C (Cao and Hegele, 2003). Four individuals in ExAC carried p.R527H (allele frequency 0.00683%); this variant is also reported in the laminopathy cohort in 19 asymptomatic heterozygous individuals and 18 patients with MADA due to p.R527H homozygosity (Novelli et al., 2002; Shen et al., 2003; Simha et al., 2003; Garavelli et al., 2009) or compound heterozygosity with V440M (Lombardi et al., 2007). Since ‘progeria patch’ variants appear to cause autosomal recessive disorders, the eight individuals in ExAC who carry such variants in heterozygosity may not manifest phenotypes. Although the frequency of the progeria-associated p.R527H allele (0.00683%) in the ExAC cohort might seem high compared to most other *LMNA* variants (only 1–2 individuals; **Figure 1A**), this allele was still rare (4 of ∼58,000 chromosomes), and might reflect population enrichment in ExAC. For example the p.R435C allele (not found in ExAC) is enriched among Caucasians (Starke et al., 2013), the p.R527H allele (4 individuals in ExAC) is enriched in Italians (Novelli et al., 2002) and the p.R527L allele (not found in ExAC) is enriched among northeastern Egyptians (Al-Haggar et al., 2012).

As noted above, only one individual in ExAC has an Ig-fold variant in the FPLD2 ‘hotspot’ (p.R482Q). Other variants linked to FPLD2 (**Figure 4B**; Burke and Stewart, 2002) or reported in metabolic syndrome patients (MetS; **Figure 4B**) are also somewhat clustered, affecting residues R439, G465, R482, K486, H506, or K515 on the Ig-fold surface (Chirico et al., 2014; Scharner et al., 2014). Three individuals in ExAC have variant p.R439C (allele frequency 0.00278%), implicated in FPLD2 and metabolic syndrome. Related to FPLD2, there is also a biochemically defined molecular feature (‘acidic patch’) required for SUMO1 modification of Ig-fold residue K486 (**Figure 4B**; Simon et al., 2013). Only one individual in ExAC had an ‘acidic patch’ variant (p.D461Y), compared to four individuals in the laminopathy database with p.D461Y (two with DCM, one with EDMD, one asymptomatic) and three with p.G465D (all FPLD2 patients). Overall, few individuals in ExAC harbored Ig-fold variants linked to FPLD2.

### Variants in the C-Terminal Region Unique to Lamin A and Its Precursor

The C-terminal ∼100 residues of prelamin A lack defined structure, but are nonetheless conserved in evolution (**Figure 5A**). The ExAC cohort included disease-associated variants p.S573L, p.G602S, p.R644C, and p.R644H, which map to this region and affect residues that are invariant or conserved, along with 27 novel variants including p.G638R (17 individuals; allele frequency 0.0146%) and p.N660D (5 individuals; allele frequency 0.00412%; **Figure 5A**). This unstructured region has many reported post-translational modifications including phosphorylation and *O*-GlcNAcylation (Simon and Wilson, 2013; Simon et al., unpublished) that would be blocked by novel variants p.S612P, p.S613P, p.S628R, and p.T643I (identified in one individual each) and novel variants p.S625C and p.S625R, identified in three individuals each (**Figure 5A**).

Strikingly, 213 individuals in the ExAC cohort have variants located within residues 620–664 (**Figure 5B**). Variants in this region might affect prelamin A processing efficiency. This prediction is based on evidence that at least 23 residues upstream of Y646 in the prelamin A substrate were required for 100% efficient cleavage by ZMPSTE24; shorter prelamin A polypeptides were cleaved inefficiently (50% uncleaved) or failed to be cleaved (**Figure 5B**; Barrowman et al., 2012). Cleavage is also reduced or abrogated by single substitutions near Y646 (pink or purple boxes, respectively, in **Figure 5B**) (Barrowman et al., 2012). Remarkably p.R644C, the most frequent ExAC variant (143 heterozygous individuals, 1 homozygous individual; allele frequency 0.1243%), adjoins the ZMPSTE24-dependent cleavage site and partially blocks prelamin A cleavage in a cell-based assay (Barrowman et al., 2012). Variant p.R644C is reported in individuals with confusingly diverse phenotypes including DCM, FPLD2 and EDMD (**Figure 1B**; Rankin et al., 2008). Novel ExAC variants in this region, particularly p.S625C and p.S625R (3 individuals each), p.G638R (17 individuals; allele frequency 0.0146%), and p.N660D (5 individuals; allele frequency 0.00412%; **Figure 5B**) deserve further study to determine whether they affect prelamin A processing. Of note, 16 of 17 individuals in ExAC with the novel p.G638R allele are African American (allele frequency 0.168% among African Americans), warranting further study of its molecular and physiological impact(s).

## Discussion

We mined DNA sequencing data from 60,706 unrelated individuals in the ExAC database to assess the prevalence of missense mutations in a single gene, *LMNA*. The resulting *LMNA* missense variants were all rare with allele frequencies ranging from 1-per-100,000 to 1-per-1,000, and were primarily heterozygous as expected (only two homozygotes: p.S625C and p.R644C; Supplementary Table 1). The ExAC assigned loss-intolerance score for *LMNA* was very high (pLI = 0.99), consistent with its functional conservation and with ExAC data for another multifunctional polymer, vimentin (*VIM*; MIM:193060), which had only two homozygous missense variants and a high loss-intolerance score (pLI = 0.96). We identified many *LMNA* variants with known or potential links to disease. One general conclusion is that the ExAC cohort included <20% of variants previously observed in laminopathy patients; ∼35 variants were represented in both the ExAC and laminopathy databases (‘shared’), whereas ∼153 known variants were not identified in ExAC, supporting the pathogenicity of variants ‘unique to laminopathy’ (**Figure 2B**). The five most frequent disease-associated variants in ExAC were p.I299V, p.S573L, p.G602S, p.R644C, and p.R644H. Further genotype/phenotype analysis is warranted, especially for variant p.G602S, a potential risk factor for Type 2 Diabetes in African Americans. Other disease-associated variants that appear to be enriched in specific ethnic groups include p.I299V in Latinos and p.R644C in South Asians and non-Finnish Europeans. Variant p.I299V is linked to FPLD2 in the laminopathy database: a puberty-onset disorder characterized by lipodystrophy, muscle hypertrophy, and insulin-resistant diabetes (Vigouroux et al., 2000; Guénantin et al., 2014), and elevated hepatic glucose production (Rizza, 2010). Variant p.I299V is also reported in one patient with DCM (Pugh et al., 2014). We identified novel variant p.G638R at higher frequency among African Americans in the ExAC cohort; we predict p.G638R likely perturbs prelamin A processing and/or an unknown function specific to mature lamin A. Interestingly p.G638R was previously identified in three patients with DCM, but classified as ‘likely benign’ in a cohort that was ∼60% European American and ∼3.2% African American (Pugh et al., 2014). These findings highlight the value of ethnically diverse cohorts such as ExAC, and the need for further molecular and physiological studies of *LMNA* variants to understand their predictive value in personalized medicine. Prediction will be especially important for dilated cardiomyopathy (DCM), where potentially pathogenic *LMNA* mutations are seen in 5.3 to 6.5% of patients (Lakdawala et al., 2012; Pugh et al., 2014) and exhibit a high incidence of phenotypic progression with adverse clinical outcomes (Kumar et al., 2016).

Our study identified novel *LMNA* variants predicted to perturb specific regions of lamin A/C polypeptides, including atomic ‘features’ on the Ig-fold surface distinct from those previously implicated in striated muscle disease, progeria or metabolic disorder. Further work to determine which interaction partners, functions or modifications are disrupted by these variants may yield biochemical insight into the functions and tissue-specific disease mechanisms of A-type lamins.

Our study has several limitations. For instance, we only assessed *LMNA* missense variants represented in ExAC, the laminopathy database and Type 2 Diabetes Knowledge Portal, and hence excluded information that may be available in other web-based or literature-based sources. Our ability to assess the potential phenotypic consequences of variants was limited by the availability of phenotypic data in ExAC. Furthermore the enrichment of certain variants may be due to population stratification within these datasets. Nevertheless the overall frequencies of certain disease-associated variants in ExAC, including p.G602S (allele frequency 0.02616%) and p.R644C (allele frequency 0.1243%) are significant because they suggest that certain *LMNA* mutations may be more common than previously recognized. These findings warrant deeper genotype/phenotype analysis to assess the full range of *LMNA* mutations, including splicing defects, as risk factors for new disease associations (e.g., sick sinus syndrome; Zaragoza et al., 2016) and as risk factors for complex traits including psychiatric disease (e.g., novel variant p.K108E) and Type 2 Diabetes. Our finding that *LMNA* variant p.G602S is overrepresented in African Americans with Type 2 Diabetes complements a much larger genome-wide meta-analysis of diabetic susceptibility genes in which individuals of African descent were insufficiently represented (Mahajan et al., 2014). However, further research is required to exclude the possibility of overrepresentation of variant alleles in certain populations independently of diabetic status. The ethnic information available in ExAC also shows clustering of certain *LMNA* variants within specific population cohorts. Deeper analysis of genomic data with more extensive information about individuals is needed to assess potential correlation with disease.

Our finding that p.G602S associates with type 2 diabetes expands the evidence that *LMNA* mutations perturb metabolism. This finding has interesting implications for the phenotype known as metabolic syndrome, which refers to multiple risk factors (abdominal obesity, elevated blood pressure, elevated fasting plasma glucose, high serum triglycerides). Individuals with three or more factors are significantly more likely to experience cardiovascular disease, stroke, and type 2 diabetes (Grundy, 2008). Other phenotypes often seen in metabolic syndrome and type 2 diabetes include increased hepatic *de novo* lipogenesis and triacylglycerol secretion (Hellerstein et al., 1996; Vedala et al., 2006). Non-alcoholic fatty liver disease is proposed to be the hepatic incarnation of metabolic syndrome (Sanders and Griffin, 2016). Lamin A variants, particularly those linked to FPDL2, can cause liver steatosis (Hooper et al., 2011). *LMNA* missense mutations were identified in two studies of metabolic syndrome patients. In particular, Decaudain et al. (2007) studied 277 patients with severe metabolic syndrome; of these, 27 individuals (10%) had a *LMNA* mutation: 17 patients had variants at Ig-fold residue R482 (the hotspot for FPLD2 mutations), and 10 patients had mutations in the lamin A head domain (p.R28W), coiled-coil ‘rod’ (p.L92F), Ig-fold (p.R439C, p.H506D) or unstructured regions in the neck or tail (p.L387V, p.S395L, p.R399H, p.L421P or T655fsX49). In a separate study of 87 ‘typical’ metabolic syndrome patients, 10 patients had perturbed lamin A/C distribution and nuclear shape defects: among these, two patients had a *LMNA* variant (p.G411D or p.G631D), and a third patient had a *ZMPSTE24* mutation and accumulated farnesylated prelamin A (Dutour et al., 2011). These two studies support the hypothesis that *LMNA* mutations in the general population contribute to metabolic disease risk.

Although specific mechanisms are not yet understood, A-type lamins influence metabolism, respiration, mitochondria, fasting insulin levels and blood glucose levels in mice (Lopez-Mejia et al., 2014), and influence the mTOR pathway, which senses nutrients including glucose and regulates catabolic pathways including autophagy (Park et al., 2009; Choi et al., 2012; Ramos et al., 2012; Shimobayashi and Hall, 2014; Cattin et al., 2015; Evangelisti et al., 2016). A-type lamins are also modified by the nutrient-responsive enzyme OGT (*O*-GlcNAc transferase), which adds a sugar, *O*-GlcNAc (β-*O*-linked *N*-acetylglucosamine) to Ser/Thr residues of target proteins (Wang et al., 2010; Bond and Hanover, 2013; Zhao et al., 2015). Several variants including p.G602S, p.G638R, and p.R644C are located within an *O*-GlcNAc-modified region (‘sweet spot’) unique to lamin A (Wang et al., 2010; Simon et al., unpublished). Further studies of human *LMNA* variants, including novel variants identified in this work, may provide insights necessary to understanding the many mechanisms and pathways by which A-type lamins influence tissue-specific signaling, chromatin organization and gene silencing in health and disease (Rodríguez and Eriksson, 2011; Conneely et al., 2012; Charar and Gruenbaum, 2017).

## Author Contributions

Conception and first draft: KW. Preparation of figures and tables: AF and TD. Data analysis: AF, TD, JJ, and KW. Intellectual input, manuscript preparation and editing: AF, TD, JJ, DV, and KW.

## Conflict of Interest Statement

The authors declare that the research was conducted in the absence of any commercial or financial relationships that could be construed as a potential conflict of interest.

## Footnotes

1 www.umd.be

2 http://www.type2diabetesgenetics.org/home/portalHome

3 http://www.grigoryanlab.org/drawcoil/

4 http://bit.ly/2hhkdOm
